# Therapeutic potential of resveratrol in Alzheimer's disease

**DOI:** 10.1186/1471-2202-9-S2-S6

**Published:** 2008-12-03

**Authors:** Valérie Vingtdeux, Ute Dreses-Werringloer, Haitian Zhao, Peter Davies, Philippe Marambaud

**Affiliations:** 1 Litwin-Zucker Research Center for the Study of Alzheimer's Disease, The Feinstein Institute for Medical Research, North Shore-LIJ, Manhasset, NY 11030, USA; 2 Department of Pathology, Albert Einstein College of Medicine, Bronx, NY 10461, USA

## Abstract

Several epidemiological studies indicate that moderate consumption of red wine is associated with a lower incidence of dementia and Alzheimer's disease. Red wine is enriched in antioxidant polyphenols with potential neuroprotective activities. Despite scepticism concerning the bioavailability of these polyphenols, *in vivo *data have clearly demonstrated the neuroprotective properties of the naturally occurring polyphenol resveratrol in rodent models for stress and diseases. Furthermore, recent work in cell cultures and animal models has shed light on the molecular mechanisms potentially involved in the beneficial effects of resveratrol intake against the neurodegenerative process in Alzheimer's disease.

## Background

Alzheimer's disease (AD) is a progressive neurodegenerative disorder leading to the most common form of dementia in elderly people. Histopathological studies of the AD brain revealed dramatic ultra-structural changes triggered by two classical lesions, the senile plaques, mainly composed of amyloid-β (Aβ) peptides, and the neurofibrillary tangles, composed of hyperphosphorylated tau proteins [1,2]. Although neurofibrillary tangles can occur independently, and cause neuronal death in frontotemporal dementia [3], the presence of both lesions in the neocortex is essential to the diagnosis of AD. The pathogenesis of the disease is complex and is driven by both environmental and genetic factors. Although most of the cases are sporadic with an obscure etiology, some forms of the disease are inherited and several genes were found to be clearly implicated in familial forms of the disease. The molecular identification and characterization of different genes associated with familial AD has provided strong support to the so-called amyloid cascade hypothesis as a causative event in the pathogenesis of AD [4]. This hypothesis states that Aβ generated from deregulated proteolysis of the amyloid precursor protein (APP) undergoes accelerated Aβ oligomerization, fibril formation, and amyloid deposition in a process that initiates the AD pathology [4].

In the past ten years, the large majority of the pharmacological research on AD has focused on understanding how Aβ is generated from APP via β- and γ-secretase cleavages, with the goal of designing specific inhibitors that will block Aβ production and the associated pathology. However, genetic and pharmacological approaches have recently demonstrated that these enzymes have additional molecular targets [5-7], and that secretase inhibitors can cause severe mechanism-based side effects *in vivo*, casting doubt over the potential of these therapeutic strategies [8]. In this context, approaches aimed at better understanding the molecular pathways involved in Aβ clearance have been gaining considerable attention over the last several years [9].

## Mechanisms of amyloid-β proteolytic clearance

A number of different receptor-mediated or endoproteolytic pathways have been implicated in the process of Aβ degradation [10]. The major enzymes responsible for Aβ degradation *in vivo *are neprilysin (NEP), endothelin-converting enzyme (ECE)-1 and ECE-2, insulin-degrading enzyme (IDE), and plasmin. NEP, IDE, and ECEs are zinc metalloendopeptidases that hydrolyze bioactive peptides and several lines of evidence support a role for these enzymes in Aβ degradation in cell cultures and animal models (reviewed in [10]).

## The plasminogen system

**Figure 1:**
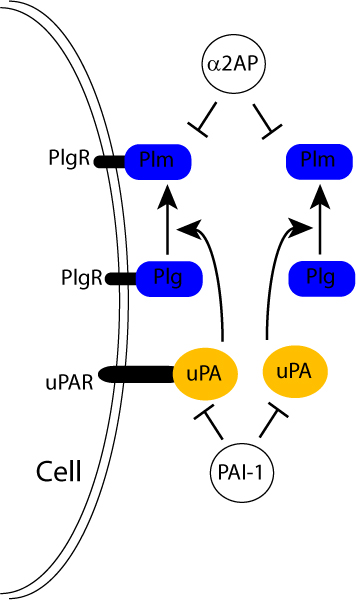
Schematic representation of the plasminogen system. The proenzyme plasminogen (Plg) is converted to the active serine protease plasmin (Plm) by the soluble or membrane bound urokinase-type plasminogen activator (u-PA). Membrane bound u-PA binds to a cellular u-PA receptor (u-PAR). Inhibition of the plasminogen system may occur at the level of the plasminogen activators by plasminogen activator inhibitors (such as PAI-1), or at the level of plasmin by α2-plasmin inhibitor (α2-PI) (also called α2-antiplasmin (α2AP)).

Plasmin is a serine protease released from the inactive zymogen plasminogen upon cleavage by the tissue-type plasminogen activator (t-PA) or by the urokinase-type plasminogen activator (u-PA) (Figure 1) [11]. The plasminogen system is negatively controlled by the action of soluble protein inhibitors that selectively bind plasminogen or plasmin. These include the plasminogen activator inhibitor-1 (PAI-1) and α2-plasmin inhibitor (α2-PI). Plasmin is involved in many pathophysiological processes primarily through its ability to degrade components of the extracellular matrix. In the brain, the plasminogen system was shown to be involved in numerous functions, including neuronal plasticity and long-term potentiation. Importantly, polymorphisms in the u-PA gene have been proposed to be associated with late-onset AD, suggesting that deregulation of the plasminogen system may be associated with the pathogenesis of the disease [12]. In addition, the t-PA-plasmin proteolytic cascade was found to promote the clearance of Aβ *in vivo *in two different mouse models of AD [13].

## Red wine intake and Alzheimer's disease

No specific environmental risk factor has been definitively identified as being associated with AD. However, the potentially important role for diet in the causation or prevention of AD is supported by several observations. For instance, there is evidence that homocysteine-related vitamins, fats, and red wine consumption have a role in the pathogenesis of AD [14]. The first study, published in 1997, reported that moderate to mild wine consumption was associated with a low risk of AD [15]. Later, a nested case-control study [16] and a cohort study [17] of individuals aged 65 years and older confirmed that intake of wine, but not other alcoholic drinks, was associated with a low risk of dementia, including AD. Furthermore, a prospective analysis of risk factors for AD in the Canadian population determined that wine consumption was the most protective variable against AD by reducing the risk of AD by 50% [18]. Interestingly, wine intake in this population was found to be even more protective than the use of nonsteroidal anti-inflammatory drugs (NSAIDs) [18]. Nevertheless, it should be noted that the notion that wine intake – and more specifically red wine intake – lowers AD risk is still controversial and remains to be clearly demonstrated. In line with the epidemiological data, however, Pasinetti and colleagues [19] have provided evidence that moderate consumption of red wine lowers Aβ levels and the associated neuropathology in the Tg2576 AD mouse model, implying that red wine intake may have a beneficial effect against AD pathology by promoting anti-amyloidogenic mechanisms.

## Bioavailability and therapeutic potential of resveratrol

Resveratrol is a phytoalexin polyphenolic compound occurring in grapes and wine. The levels of resveratrol found in wine vary greatly, but is generally more abundant in red grapes and red wine. Numerous studies performed *ex vivo *and in animal models have provided information on the absorption, metabolism, and consequent bioavailability of this polyphenol [20]. The oral bioavailability of resveratrol is low due to rapid excretion and extensive metabolism into various glucuronide and sulfate conjugates of unknown potential biological activities. The major metabolites identified in the urine in human after oral dosing of synthetic resveratrol are: resveratrol monosulphate, two isomeric forms of resveratrol monoglucuronide, dihydroresveratrol monosulphate, and dihydroresveratrol monoglucuronide. Total sulphate conjugates account for more than one-third of the metabolites in the urine and total glucuronide conjugates represent about 20% [21]. These pharmacokinetic studies cast doubt on the therapeutic potential of unmodified resveratrol. Nevertheless, *in vivo *data from several studies have clearly demonstrated in various organisms that resveratrol intake has protective properties against multiple illnesses, including cancer, cardiovascular disease, and ischemia, and was also found to confer resistance to stress and to extend life span [22]. More recently, concordant studies have also revealed the beneficial effect of resveratrol *in vivo *on energy metabolism in diseases such as diet-induced obesity, insulin resistance, or aging-related syndromes [23-25].

## The antioxidant functions of resveratrol

Resveratrol is believed to afford strong antioxidant functions *in vitro *and in cell culture models and, therefore, to contribute to the cardio-protective, anti-inflammatory, and neuroprotective properties of red wine intake [26]. Several lines of evidence underline the antioxidant and neuroprotective effects of resveratrol. The polyphenol was found to prevent membrane lipid peroxidation, internalization of oxidized lipoprotein, and to reduce the toxic effects of reactive oxygen intermediates in cultured cell lines [27,28]. In addition, resveratrol delays Aβ-induced toxicity in different neuronal culture models [29-31]. Nevertheless, the mechanism by which resveratrol is able to achieve these effects remains to be clearly defined. Independent biochemical approaches have, however, demonstrated that the polyphenol has potent anti-amyloidogenic and anti-fibril effects *in vitro *[32-35], suggesting that resveratrol may act as an antioxidant by preventing the formation of toxic Aβ oligomers and protofibrillar intermediates.

## Resveratrol, SIRT1, and amyloid pathology

Recent data provide interesting insights into the effect of resveratrol on longevity and its potential protective role in age-related human diseases, including AD. Resveratrol mimics caloric restriction by extending the lifespan of different organisms via activation of deacetylases from the sirtuin family [36]. Indeed, resveratrol binds to and activates the deacetylase activity of several sirtuin members, including the mammalian ortholog, SIRT1, by lowering the Michaelis constants of these enzymes [37]. Sirtuins are nicotinamide adenine dinucleotide-dependent deacetylases required for the increased longevity due to caloric restriction in yeast, *Drosophila*, and mice [36,38]. Sirtuins deacetylate and control the activity of several transcription factors, including peroxisome proliferator-activated receptor (PPAR)γ cofactors, through a mechanism that regulates lifespan in mice [39,40]. Importantly, Kim and colleagues [41] recently reported that intracerebroventricular injection of resveratrol reduced neurodegeneration in the hippocampus and prevented learning impairment in the p25 transgenic AD mouse model by a mechanism that may involve a decrease in the acetylation of known SIRT1 substrates, for example, peroxisome proliferator-activated receptor gamma coactivator (PGC-1)α and p53. In addition, caloric restriction was found to attenuate amyloid deposition and Aβ-associated neuropathology in different animal models [42,43], and SIRT1 activation may contribute to the anti-amyloidogenic properties of this particular intervention [44,45]. In line with these studies, resveratrol appears to be protective against the dysregulation of energy homeostasis observed in mouse models for metabolic syndromes by a mechanism implicating the activation of SIRT1, PGC-1α, and the energy sensor protein kinase AMPK (AMP-activated protein kinase) [23-25].

## Resveratrol, SIRT1, and plasmin activation

Our recent work indicates that resveratrol reduces Aβ accumulation in cell cultures [46]. Resveratrol does not inhibit Aβ production, since it has no effect on the Aβ-producing enzymes β- and γ-secretases, but promotes instead the proteolytic clearance of Aβ by a mechanism that does not implicate NEP, ECE-1 and ECE-2, or IDE [46]. Several groups have obtained evidence that resveratrol treatment increases the expression of the plasminogen activators t-PA and u-PA, suggesting that resveratrol could lead to plasminogen endoproteolysis and plasmin activation (Figure 1) [47,48]. Additional evidence indicates that plasminogen activation is positively controlled by PPARγ that appears to be a transcriptional target of SIRT1 [39,40,49]. This raises the intriguing possibility that resveratrol controls plasmin activation by promoting SIRT1-mediated transcription. This last observation may further explain the anti-amyloidogenic effect of PPARγ activation recently shown by several groups, including our own [50-53]. Additional observations argue against a direct effect of resveratrol on a protease degrading Aβ, but instead point toward the implication of a transcriptional mechanism in the regulation of Aβ degradation. There is, indeed, no data indicating that resveratrol binds to and directly stimulates a known Aβ-degrading protease. Moreover, the long incubation periods required for resveratrol action [46] clearly suggest the implication of gene expression regulation in the mechanism of Aβ clearance. Therefore, it is possible that resveratrol acts by interacting with SIRT1-mediated transcription in a pathway of Aβ degradation.

## Conclusion

Although unmodified resveratrol appears to have a weak bioavailability, several studies have clearly demonstrated the *in vivo *neuroprotective properties of the red wine-derived polyphenol, strongly supporting the notion that natural metabolites of resveratrol may have biological activities. Furthermore, recent findings have shed light on the potential role of resveratrol in transcription- and degradation-dependent anti-amyloidogenic mechanisms, suggesting that natural metabolites or potent synthetic analogues of resveratrol have a therapeutic potential in AD.

## List of abbreviations used

Aβ: amyloid-β; AD: Alzheimer's disease; APP: amyloid precursor protein; ECE: endothelin-converting enzyme; IDE: insulin-degrading enzyme; NEP: neprilysin; PGC: peroxisome proliferator-activated receptor gamma coactivator; PPAR: peroxisome proliferator-activated receptor; t-PA: tissue-type plasminogen activator; u-PA: urokinase-type plasminogen activator.

## Competing interests

The authors declare that they have no competing interests.

## Authors' contributions

PM, VV, UDW, HZ and PD conceived the project and performed the work. PM wrote the manuscript.
